# Differential vulnerability of adult neurogenic niches to dosage of the neurodevelopmental-disorder linked gene *Foxg1*

**DOI:** 10.1038/s41380-022-01497-8

**Published:** 2022-03-22

**Authors:** Iris Schäffner, Marie-Theres Wittmann, Tanja Vogel, D. Chichung Lie

**Affiliations:** 1 Institute of Biochemistry, Emil Fischer Center, Friedrich-Alexander Universität Erlangen-Nürnberg, Erlangen, Germany; 2 Institute of Anatomy and Cell Biology, Department of Molecular Embryology, Faculty of Medicine, Albert-Ludwigs-University Freiburg, Freiburg, Germany; 3 Center for Basics in NeuroModulation (NeuroModul Basics), Medical Faculty, Albert-Ludwigs-University Freiburg, Freiburg, Germany

**Keywords:** Neuroscience, Stem cells

## Abstract

The transcription factor FOXG1 serves pleiotropic functions in brain development ranging from the regulation of precursor proliferation to the control of cortical circuit formation. Loss-of-function mutations and duplications of *FOXG1* are associated with neurodevelopmental disorders in humans illustrating the importance of FOXG1 dosage for brain development. Aberrant FOXG1 dosage has been found to disrupt the balanced activity of glutamatergic and GABAergic neurons, but the underlying mechanisms are not fully understood. We report that FOXG1 is expressed in the main adult neurogenic niches in mice, i.e. the hippocampal dentate gyrus and the subependymal zone/olfactory bulb system, where neurogenesis of glutamatergic and GABAergic neurons persists into adulthood. These niches displayed differential vulnerability to increased FOXG1 dosage: high FOXG1 levels severely compromised survival and glutamatergic dentate granule neuron fate acquisition in the hippocampal neurogenic niche, but left neurogenesis of GABAergic neurons in the subependymal zone/olfactory bulb system unaffected. Comparative transcriptomic analyses revealed a significantly higher expression of the apoptosis-linked nuclear receptor *Nr4a1* in FOXG1-overexpressing hippocampal neural precursors. Strikingly, pharmacological interference with NR4A1 function rescued FOXG1-dependent death of hippocampal progenitors. Our results reveal differential vulnerability of neuronal subtypes to increased FOXG1 dosage and suggest that activity of a FOXG1/NR4A1 axis contributes to such subtype-specific response.

## Introduction

FOXG1 (Forkhead box G1; brain factor 1; BF-1) is an evolutionary conserved transcription factor with pleiotropic functions in forebrain development including early territorial specification of the telencephalon, regulation of dorsal telencephalic precursor proliferation and apoptosis, migration of neuronal precursors, and neural circuit development [1–8]. FOXG1’s critical and non-redundant role in forebrain development is underlined by the finding that mutations in the *FOXG1* gene cause *FOXG1* syndrome—a neurodevelopmental disorder featuring seizures, intellectual disability, limited communication skills, and autistic behavior. Interestingly, heterozygote loss-of-function mutations as well as gene duplications have been linked to *FOXG1* syndrome illustrating the importance of FOXG1 dosage for brain development [9–20]. Analyses of patient-derived induced pluripotent stem cell (iPSC) models also suggested dysregulated FOXG1-signaling as a pathophysiological contributor to idiopathic Autism Spectrum Disorder (ASD). Mariani and colleagues described increased FOXG1-signaling in iPSC-derived organoids from four patients with idiopathic macrocephalic ASD [21], and Marchetto and colleagues found increased FOXG1 expression in iPSC-derived neural precursor cells from seven subjects with idiopathic macrocephalic ASD [22]. Notably, these studies also found an imbalance in the activity of excitatory and inhibitory neurons in their respective iPSC models, which supports the emerging hypothesis that disruption of the synaptic excitation and inhibition (E/I) balance is involved in the pathophysiology of ASD [23, 24]. In an elegant set of experiments, Miyoshi and colleagues recently provided direct evidence for a critical role of FOXG1 in controlling the E/I balance in neural circuits in vivo [25]. Using transgenic mouse models for FOXG1 loss- and gain-of-function, this group demonstrated that concurrent dysregulation of FOXG1 in excitatory and inhibitory neurons during an early postnatal period transiently disrupts E/I balance in cortical circuits and causes ASD-like behavioral phenotypes.

In this study we aimed to compare in vivo impact of increased FOXG1 dosage on neurogenesis of GABAergic and glutamatergic neurons using neurogenesis in the subependymal zone (SEZ)/olfactory bulb (OB) system and in the dentate gyrus (DG) of the adult murine brain as model systems. Neural stem/progenitor cells (NSPCs) in the SEZ of the lateral ventricles generate mainly GABAergic inhibitory neurons that migrate through the rostral migratory stream (RMS) to integrate into the OB network [26]. In contrast, NSPCs residing at the border between the hilus and granule cell layer (GCL) of the DG of the hippocampal formation exclusively give rise to glutamatergic excitatory dentate granule neurons [27, 28]. We overexpressed murine FOXG1 in proliferating progenitor cells of both neurogenic niches in vitro and in vivo. Adult-born cells of the DG overexpressing FOXG1 showed a severe survival defect and surviving cells failed to develop the generic morphological characteristics and marker expression of glutamatergic DG neurons. In contrast, the GABAergic progeny of progenitor cells in the SEZ/OB system was unaffected by the overexpression of FOXG1. Transcriptomic analysis uncovered a significantly higher expression of the orphan nuclear receptor *Nr4a1* in FOXG1-overexpressing hippocampal NSPCs compared to their SEZ counterparts; whereas, inhibition of NR4A1 strongly reduced cell death of hippocampal NSPCs. These data provide in vivo evidence for the differential sensitivity of GABAergic and glutamatergic neurogenesis to increased FOXG1 levels and suggest differential activity of the nuclear receptor NR4A1 as a candidate pathway mediating differential vulnerability in neurogenesis of distinct neuronal subtypes in adult mice.

## Materials and methods

### Contact for reagent and resource sharing

Further information and requests for reagents may be directed to, and will be fulfilled by the corresponding author, DCL (chi.lie@fau.de).

### Experimental model and subject details

All experiments were carried out in accordance with the European Communities Council Directive (86/609/EEC) and were approved by the Government of Upper Bavaria or the Regierungspräsidium Freiburg (X-17/03S).

For all in vivo and in vitro experiments, 8-week-old female C57BL/6J mice from Charles River were used. Mice were group housed in standard cages under a 12 h light/dark cycle with ad libitum access to water and food. Mice in exercise conditions had ad libitum access to running wheels 7 days prior retroviral injections.

FoxG1^Cre/+^ mice were obtained and genotyped as described before [29, 30].

## Method details

### In vitro assays

#### Neural stem/progenitor cell (NSPC) isolation

Female 8-week-old C57BL/6J mice were sacrificed via cervical dislocation, the brains were extracted from the cranial cavity and kept in ice-cold 1xHBSS. The DG and SEZ were microdissected and collected each in ice-cold 1xHBSS [31]. NSPCs were subsequently isolated with the MACS neural tissue dissociation kit according to manufacturer’s protocol (MACS Miltenyi Biotec, Cat# 130-092-628). NSPCs were kept under proliferative conditions in DMEM F12 Glutamax (GIBCO, Cat# 10565018) medium with 1xNeurobrew-21 (MACS Miltenyi Biotec, Cat# 130-093-566), 1xPSF (GIBCO, Cat# 15240062), 8 mM HEPES, 10 ng/ml EGF (Peprotech, Cat# AF-100-15) and 10 ng/ml FGF (Peprotech, Cat# AF-100-18B). NSPCs were maintained as free-floating neurospheres as described before [32].

#### Retroviral transduction of NSPCs

NSPCs, dissociated into single cells, were seeded onto tissue culture plates or glass cover slips coated with PDL(Poly-D-Lysine)/Laminin. 24 h later, adherent NSPCs were transduced with a green fluorescent protein (GFP)-encoding control MML-retrovirus or an MML-retrovirus bi-cistronically encoding for FOXG1 and GFP. Overexpression of FOXG1 was validated via immunocytochemistry (ICC) and western blot analyses.

#### Proliferation assays

To measure self-renewal capacity of NSPCs, a single cell neurosphere assay was applied. NSPCs, dissociated into single cells, were seeded in a density of 1 cell per well into the wells of 60-well Nunc^®^ MicroWell^®^ MiniTrays (Sigma, Cat# M0815) in growth medium. The number of formed neurospheres was determined under a brightfield microscope 7 days after seeding.

To measure proliferation, NSPCs dissociated into single cells were seeded in a density of 100,000 cells per well into the wells of 24-well-plates with PDL/Laminin-coated cover slips (Greiner Bio-one, Cat# 662160) in growth medium. 24 h later, NSPCs were treated with 5 mM BrdU (5-bromo-2'-deoxyuridine) for 3 h and fixated with 4% PFA for 10 min at room temperature. NSPCs were washed with PBS and stored at 4 °C until further use.

#### Viability and cell death assays

To measure viability, NSPCs dissociated into single cells were seeded in a density of 500,000 cells per well of a 24-well tissue culture plate in growth medium and treated as indicated. 15 µl of the cell suspension were mixed with 15 µl of Trypan blue. The number of dead and living cells was determined under a fluorescent microscope with a Neubauer counting chamber. The cell death rate was calculated as the ratio of dead cells per total number of cells (dead + living cells).

To analyze cell death in vitro, NSPCs were treated as indicated and the ApopTag^®^ Red In Situ Apoptosis Detection Kit was used according to manufacturer’s protocol (Merck Millipore, Cat# S7165).

#### Differentiation analysis

NSPCs were dissociated into single cells and seeded in a density of 100,000 cells per well on PDL/Laminin-coated glass cover slips into 24-well-plates in growth medium. After 24 h, growth medium was replaced by differentiation medium (growth medium without EGF and FGF) and cells differentiated for 6 days. After fixation with 4% PFA for 10 min at room temperature, cells were washed with PBS and stored at 4 °C until further use.

#### Immunocytochemistry

ICC on PFA fixed NSPCs was performed as described before [32]. Primary antibodies were incubated over night at 4 °C and visualized with Cy3/Cy5-conjugated (Jackson Laboratories) or Alexa488-conjugated (Invitrogen) secondary antibodies (all 1:500). For 5-bromo-2'-deoxyuridine (BrdU) staining, cells were incubated in 2 N HCl for 10 min at 37 °C prior the staining procedure. The following primary antibodies were used for ICC: BrdU, Bio-Rad Cat# MCA2060 (1:500); DCX, Santa Cruz Biotechnology Cat#8066 (1:500); GFAP, Sigma Cat#G3893 (1:1000); GFP, Aves Cat#GFP-1020 (1:1000); FOXG1, Abcam Cat# ab18259 (1:500); FOXG1, Abcam Cat#ab3394 (1:500); NR4A1, Abcam Cat#ab153914 (1:500).

#### Fluorescent signal intensity measurements

To analyze the expression levels of FOXG1 in retroviral transduced (CAG-GFP, CAG-FOXG1-IRES-GFP) cells in vitro and in vivo, the mean pixel value (mean grey value) of the fluorescent signal within a cell was determined with Fiji ImageJ.

To analyze the expression levels of NR4A1 in vitro, Corrected Total Cell Fluorescence (CTCF) was calculated; the fluorescence’s level within the area (cytoplasm or nucleus) of one cell was determined using Fiji ImageJ as previously described [33].

#### SDS-PAGE and western blotting

For SDS-PAGE and western blot analysis NSPCs were lysed in RIPA buffer (1% Nonidet P40, 0.1% SDS, 0.5% sodium deoxycholate, 50 mM Tris, 150 mM NaCl, 1 mM EDTA, complemented with protease and phosphatase inhibitors) for 20 min on ice. Total protein containing supernatant of the lysate was obtained by centrifugation at 2000 *g* for 10 min at 4 °C. Total protein was determined using Pierce BCA protein assay (Thermo Scientific, Cat# 23225). 30 µg of total protein were separated on 4–12% Bis-Tris gels (Life Technologies, Cat# NP0322BOX) and transferred to a PVDF membrane (Immobilon-P, Millipore, Cat# IPVH00010). Membranes were washed with 0.1% Tween 20 in PBS (PBS-T) and blocked for 1 h at room temperature in 1% BSA in PBS-T. Incubation with primary antibodies was performed overnight at 4 °C. After washing with PBS-T, membranes were incubated with HRP-labeled secondary antibodies (Jackson Laboratories) for 1 h at room temperature (all 1:10,000). Immunoblots were analyzed with the FusionFX (Peqlab, Erlangen, Germany) and quantified using the Bio1D software (Vilber Lourmat, Eberhardzell, Germany). The following primary antibodies were used for western blot analysis: ACTIN, Millipore Cat# MAB1501 (1:2,000), FOXG1, Abcam Cat# ab18259 (1:500); NR4A1, Abcam Cat#ab13851 (1:500).

#### RNA isolation

Total RNA isolation from NSPCs was performed using a protocol combining TRIzol^®^ Reagent (Invitrogen, Cat# 15596026) and the Rneasy Mini Kit (QIAGEN, Cat# 74104). Pelleted adult NSPCs (aNSPCs) were homogenized in 1 ml of TRIzol^®^ Reagent and incubated for 5 min at RT. 200 µl of Chloroform was added and mixed vigorously. After 2–3 min at RT, samples were centrifuged at 10,000 *g* for 20 min at 4 °C. The aqueous phase was transferred to a new sterile RNA-free tube, mixed with an equal volume of 100% RNA-free EtOH and loaded onto a Rneasy Mini column. The further RNA isolation was performed according to the Rneasy Mini Kit manufacturer’s protocol (QIAGEN, Cat# 74104).

#### cDNA preparation

For cDNA preparation the RevertAid First Strand cDNA Synthesis Kit was used according to manufacturer’s protocol (Thermo Scientific, Cat# K1621).

#### qRT-PCR

Quantitative Real-Time PCR was performed on a Roche Light Cycler (384-well format) using Power SYBR Green PCR Master Mix (Thermo Scientific, Cat# 4368577) according to the manufacturer’s instructions. For each experimental group the cDNA from four independent biological replicates was used. To quantify the changes in mRNA expression, the 2^−ΔΔCT^ method was used [34]. Mean CT values of each target gene were normalized to the mean of the mean CT values of three housekeeping genes (GAPDH, RPL13a, RPL27). Fold changes were calculated by normalizing each ΔΔCT value to the control ΔΔCT values. Following qRT-PCR oligonucleotide sequences were used:

*Ccnb2*_F CGACGGTGTCCAGTGATTTG, *Ccnb2*_R TCGGCTGCTTGTTGACATTT; *Cdk1*_F GGGCACTCCTAACAACGAAG, *Cdk1*_R CCAGAGATTCGTTTGGCAGG; *Nr4a1*_F TTCGGCGTCCTTCAAGTTTG, *Nr4a1*_R AAGTTGGGTGTAGATGGCGA; *Nupr1*_F CTTGCCACCAACAGCCAAC, *Nupr1*_R CGGTTGGTATTGGCAGCAG.

#### Bulk RNA sequencing (RNA-seq)

For Bulk RNA-seq DG and SEZ NSPCs were seeded in PDL/Laminin-coated 6-well plates in a density of 1 million cells per well. After 24 h DG and SEZ NSPCs were transduced either with a GFP-expressing control retrovirus (CAG-GFP) or a FOXG1-overexpressing retrovirus (CAG-FOXG1-IRES-GFP). Four independent biological replicates were used. DG and SEZ NSPCs were harvested 3 days after retroviral transduction and total RNA isolated as described in the paragraph “RNA isolation” above. 2 µg total RNA of each sample was sent for Bulk RNA-seq (GENEWIZ Germany GmbH). After RNA quality control by GENEWIZ, libraries for sequencing were prepared using Illumina^®^, strand-specific RNA-seq with PolyA selection and sequenced using the Illumina^®^ NovaSeq^TM^ platform (2 × 150 bp configuration, 20 M paired-end reads per sample). We up-loaded the raw data to the GALAXY web platform and analyzed the data utilizing the public server at usegalaxy.org [35]. The following workflow was implemented: After quality filtering with FASTQ Trimmer, we aligned the reads to the mouse reference genome Ensembl GRCm39, release 104 [36]. We obtained read counts per gene using htseq-count and Ensembl gff3 annotation file [37]. DESeq2 was used for differential expression analysis [38]. Genes were considered differentially expressed if the Benjamini–Hochberger adjusted *p*-value was less than 0.01. Genes with a log2 fold change >1.5 or < −1.5 were considered for Gene Ontology (OG) term analysis. GO term and KEGG pathway analyses were performed using Database for Annotation, visualization, and Integrated Discovery (DAVID; david.ncifcrf.gov) [39, 40]. RNA-seq data can be accessed in the NCBI gene expression and hybridization array data repository (GEO) database and have the accession number GSE185315.

### Tissue processing

Adult animals were sacrificed by CO_2_, transcardially perfused with 100 ml phosphate-buffered saline (PBS, pH 7.4) followed by 100 ml 4% paraformaldehyde (PFA, pH 7.4, Roth, Cat# 0335) in PBS at a rate of 20 ml/min and brains were post fixed in 4% PFA for 3 h at room temperature. Subsequent transfer to a 30% sucrose solution allowed for coronal and sagittal brain sectioning at 50 µm thickness using a sliding microtome (Leica Microsystems, Wetzlar, Germany). E13.5 mouse brains were harvested, treated and sliced on a cryotome (Leica Microsystems, Wetzlar, Germany) as described previously [41].

### Histology and counting procedures

Immunofluorescent stainings of free-floating 50 µm sections of adult mice and 10 µm sections on laminated object slides of embryonic mice were performed as previously described [32, 41]. The following primary antibodies were used for immunohistochemistry: cFOS, Santa Cruz Biotechnology Cat#sc271243 (1:500); CLEAVED CASPASE 3, Cell Signaling Technology Cat#9661 (1:500); DCX, Santa Cruz Biotechnology Cat#8066 (1:500); FOXG1, Abcam Cat# ab18259 (1:500); FOXG1, Abcam Cat#ab3394 (1:500); GAD67, Millipore Cat#MAB5406 (1:500); GFAP, Sigma Cat#G3893 (1:1000); GFP, Aves Cat#GFP-1020 (1:1000); MAP2(2a + 2b), Sigma Cat#M1406 (1:500); NEUN, Chemicon Cat#IHCR1001-6 (1:500); OLIG2, Millipore Cat#AB9610 (1:500); PROX1, Millipore Cat# AB5475 (1:500); RFP, ChromoTek Cat#5F8-100 (1:1,000); SOX2, Santa Cruz Biotechnology Cat#sc17320 (1:250); TBR2, Abcam Cat#ab23345-100 (1:500); TH, Protos Biotech Cat#CA-101 (1:500); VGLUT1, Synaptic Systems Cat#135302 (1:500). Primary antibodies were visualized with Cy3/Cy5-conjugated (Jackson Laboratories) or Alexa488-conjugated (Invitrogen) secondary antibodies (all 1:500).

Single plane images and z-stacks were taken either with confocal microscopes (Leica SP5 or a Zeiss LSM 780) each equipped with four laser lines (405, 488, 559 and 633 nm) and ×63, ×40 and ×25 objective lens or an ApoTome fluorescent microscope (Zeiss ApoTome.2). Images were processed using Fiji ImageJ [42].

### Retrovirus preparation and stereotactic injections

The CAG-GFP and CAG-RFP plasmids have been previously described [43]. For generation of the CAG-FOXG1-IRES-GFP the *GFP* coding sequence of the CAG-GFP-IRES-GFP retroviral plasmid was replaced with the murine *Foxg1* sequence. MML-retroviruses were generated as previously described [44]. Viral titers were ~2 × 10^8^ colony-forming units (cfu) ml^−1^.

Stereotactic injections in 8-week-old female C57BL/6J mice were performed as described previously [32]. Injection coordinates from bregma were −1.9 mm anterior/posterior, ±1.6 mm medial/lateral, −1.9 mm dorsal/ventral from dura for DG injections and/or +2.3 mm anterior/posterior, ±0.8 mm medial/lateral, −2.9 mm dorsal/ventral from dura for SEZ/RMS injections.

## Quantification and statistical analysis

### Expression analysis of stage-specific markers

For the quantifications of immunohistochemical stainings at least 50 cells per animal and marker in 5–6 sections containing the DG and/or at least 3 sections containing the SEZ, RMS and OB from at least 3 different animals were analyzed. No statistical methods were used to predetermine sample sizes, but our sample sizes were similar to those reported in previous publications [32]. The quantification of performed immunohistochemical stainings was performed blinded.

### Statistical analysis

The statistical analysis was performed using GraphPad Prism (version 9). Significance levels were assessed using unpaired Welch’s *t*-test with unequal variances if the data followed a normal distribution according to the Shapiro–Wilk test. Otherwise, the non-parametric Mann Whitney test was performed. To assess significance for experiments with two independent variables a two-way ANOVA (analysis of variance) was performed. Differences were considered statistically significant at *p < 0.05, **p < 0.01 and ***p < 0.001. All data are presented as mean ± SEM (standard error of the mean).

## Results

### FOXG1 is expressed in the neurogenic niches in the adult murine brain

We first determined the expression pattern of FOXG1 within the neurogenic lineage of the adult DG and the adult SEZ/RMS/OB in vivo. For validation of the specificity of the applied FOXG1 antibodies we stained the cortex of E13.5 (Embryonic day 13.5) FOXG1 WT and KO littermates (Fig. S1A–D). WT cortex showed for both FOXG1 antibodies a clear mainly nuclear staining, while no staining beyond background was detectable in the cortex of E13.5 FOXG1 KO, verifying the specificity of the applied FOXG1 antibodies.

**Fig. 1:**
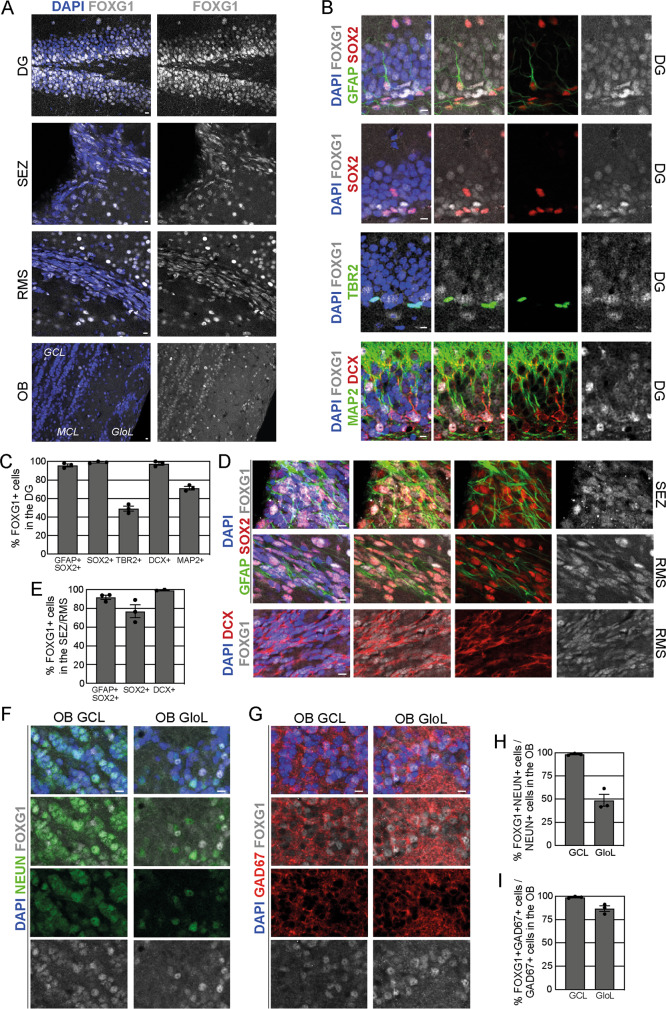
FOXG1 expression in adult neurogenic niches. **A** Confocal images of the adult murine DG, SEZ, RMS and OB at P56 with immunohistochemistry for FOXG1 (gray) and DAPI (blue). Scale bars = 10 µm. GCL granule cell layer, MCL mitral cell layer, GloL glomerular layer. **B** Confocal images of the adult DG (P56) with immunohistochemistry for FOXG1 (gray) together with stage-specific markers. Immunohistochemistry for SOX2 (red) and GFAP (green) was used to detect adult radial glia-like neural stem cells, SOX2 (red) for progenitor cells, TBR2 (green) for transient amplifying progenitors (TAPs), DCX (red) for neuroblasts and immature neurons and MAP2 (green) for mature neurons. DAPI in blue. Scale bar = 10 µm. **C** Quantitative analyses showed that FOXG1 is co-expressed in nearly all SOX2 + GFAP + radial glia-like neural stem cells and SOX2+ progenitor cells in the adult DG. Only 50% of the TBR2 + TAPs expressed FOXG1, while nearly all DCX+ neuroblasts and immature neurons and ca. 70% of all mature MAP2+ neurons expressed FOXG1. 50–100 cells/animal analyzed; *n* = 3. **D** Confocal images of the adult SEZ and RMS (P56) with immunohistochemistry for FOXG1 (gray) together with stage-specific markers. Immunohistochemistry for SOX2 (red) and GFAP (green) was used to detect adult neural stem cells, SOX2 (red) for progenitor cells, DCX (red) for neuroblasts and immature neurons. DAPI in blue. Scale bar = 10 µm. **E** Quantitative analyses showed co-expression of FOXG1 with SOX2 + GFAP + neural stem cells, SOX2+ progenitors and DCX+ neuroblasts in the adult SEZ/OB. 50–200 cells/animal analyzed; *n* = 3. **F**, **G** Confocal images of the granule cell layer (GCL) and the glomerular layer (GloL) of the adult OB with immunohistochemistry for FOXG1 (gray), the neuronal marker NEUN (green) and GAD67 (red) for GABAergic neurons. **H**, **I** Quantifications showed that nearly all NEUN+ cells in the GCL and 50% in the GloL expressed FOXG1 in the adult OB. Nearly all GAD67 + GABAergic neurons were positive for FOXG1 in the GCL and ca 90% in the GloL in the adult OB. 100–250 cells/animal analyzed; *n* = 3. Data represented as mean ± SEM.

Immunohistochemistry of the adult mouse brain showed that FOXG1 protein is expressed both in the subgranular zone and the GCL of the DG and in the SEZ, RMS and the layers of the OB in young adult (Postnatal day 56; P56) wildtype mice (Fig. 1A). Co-immunohistochemical stainings with stage-specific markers detected a dynamic expression pattern of FOXG1 in the neurogenic lineage of the adult DG (Fig. 1B). We used the combination of SOX2 and GFAP (Glial Fibrillary Acid Protein) to identify the radial glia-like neural stem cells. FOXG1 is expressed by the vast majority of SOX2 + GFAP + radial glia like neural stem cells and SOX2+ progenitor cells in the adult DG, while only 50% of the TBR2+ transient amplifying progenitor cells were positive for FOXG1 (Fig. 1B, C). Following this drop in expression, most of the DCX+ neuroblasts and immature neurons expressed FOXG1 again (Fig. 1B, C). Approximately 70% of the MAP2+ mature neurons in the DG co-expressed FOXG1 (Fig. 1B, C). Co-immunohistochemical stainings revealed a more constant expression of FOXG1 protein in the adult SEZ and RMS neurogenic lineage (Fig. 1D, E). FOXG1 is expressed in >90% of SOX2 + GFAP + neural stem cells, in about 80% of the SOX2+ progenitor cells of the SEZ/RMS and in virtually all of the migrating Doublecortin-positive (DCX+) neuroblasts in the RMS (Fig. 1D, E). We used NEUN as a general neuronal marker to analyze neuronal FOXG1 expression in the GCL and the glomerular layer (GloL) of the OB. Next to all NEUN+ neurons of the GCL and around 50% of the NEUN+ neurons of the GloL were positive for FOXG1 (Fig. 1F, H). Adult neurogenesis in the SEZ produces mainly GABAergic neurons. Using GAD67 (Glutamic Acid Decarboxylase 67) as a marker for GABAergic neurons we found that almost all GAD67 + GABAergic neurons in the GCL and over 80% of the GAD67+ cells in the GloL co-express FOXG1 (Fig. 1G, I). Intensity measurements of the FOXG1 fluorescent signal also indicated that FOXG1 levels in the DG neurogenic lineage were lowest in TBR2+ transient amplifying progenitor cells, whereas FOXG1 expression levels in the SEZ/RMS/OB neurogenic lineage were more uniform (Fig. S1E).

### Increased FOXG1 dosage impairs DG adult NSPC but not SEZ adult NSPC function in vitro

**Fig. 2:**
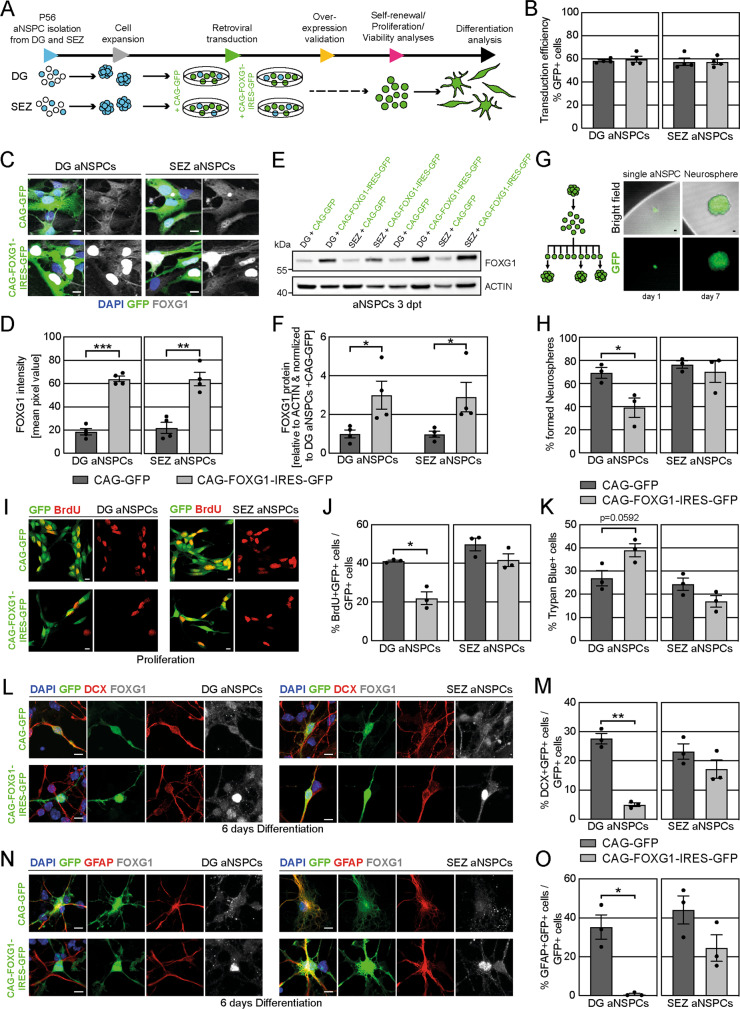
FOXG1 overexpression diminishes the self-renewal, proliferation and differentiation capacity of DG aNSPCs in vitro. **A** Experimental paradigm used to analyze FOXG1 dosage in aNSPCs in vitro. aNSPCs were isolated from the DG and the SEZ of wildtype mice at P56. Cells were expanded and transduced with either a GFP-encoding control retrovirus or a FOXG1-IRES-GFP-encoding retrovirus. Following confirmation of FOXG1 overexpression, cells were analyzed for their self-renewal, proliferation, viability and differentiation behavior. **B** Analyses of retroviral transduction efficiency showed an average of 60% retroviral transduction for the applied retroviruses and cell types. *n* = 4 biological replicates/group. **C** Validation of the FOXG1 overexpression in DG and SEZ aNSPCs via immunocytochemistry. FOXG1 in gray. DAPI in blue. Scale bar = 10 µm. **D** Mean fluorescent FOXG1 signal intensity was 3-fold increased in DG and SEZ aNSPCs in cells transduced with the CAG-FOXG1-IRES-GFP retrovirus. *n* = 4 biological replicates/group. **E** Western blot analysis of protein lysates of DG and SEZ aNSPCs transduced with either CAG-GFP control or the FOXG1 overexpression (CAG-FOXG1-IRES-GFP) retrovirus. An antibody against FOXG1 was applied. ACTIN was used as endogenous loading control. For quantification in **F**, protein amounts relative to ACTIN were subsequently normalized to DG aNSPCs transduced with the CAG-GFP control retrovirus. dpt days post transduction. **F** Retroviral FOXG1 overexpression led to an average 3-fold increase of FOXG1 protein in DG and SEZ aNSPCs. *n* = 4 biological replicates/group. **G**, **H** Experimental scheme and quantifications of the single cell neurosphere assay to assess the self-renewal potential of aNSPCs. Fluorescence images of a single seeded aNSPC and a neurosphere 7 days after seeding onto miniwells. The self-renewal potential was significantly reduced in FOXG1-overexpressing aNSPCs from the DG. FOXG1 overexpression did not alter the self-renewal capacity of SEZ aNSPCs. *n* = 3 biological replicates/group. Scale bar = 10 µm. **I** Confocal images of proliferating aNSPCs transduced either with a CAG-GFP control retrovirus or a CAG-FOXG1-IRES-GFP retrovirus. Immunocytochemistry for BrdU in red. Scale bar = 10 µm. **J** Proliferation analysis via BrdU incorporation revealed a significantly reduced proliferation in DG aNSPCs due to FOXG1 overexpression. Proliferation of SEZ aNSPCs was unaffected by FOXG1 overexpression. *n* = 3 biological replicates/group. **K** Viability assay via Trypan blue incorporation showed a trend towards increased cell death in DG aNSPCs due to FOXG1 overexpression. The viability of SEZ aNSPCs was not affected by FOXG1 overexpression. *n* = 3 biological replicates/group. **L** Confocal images of 6 days differentiated aNSPCs from DG and SEZ retrovirally transduced with either a CAG-GFP control retrovirus or a CAG-FOXG1-IRES-GFP retrovirus. Immunocytochemistry for DCX (neuronal marker) in red and FOXG1 in gray. DAPI in blue. Scale bar = 10 µm. **M** Quantifications determined that FOXG1 overexpression severely diminished neuronal differentiation of DG aNSPCs while the SEZ aNSPCs were unaffected compared to the CAG-GFP control cells. *n* = 3 biological replicates/group. **N** Confocal images of 6 days differentiated aNSPCs from DG and SEZ retrovirally transduced with either a CAG-GFP control retrovirus or a CAG-FOXG1-IRES-GFP retrovirus. Immunocytochemistry for GFAP (astrocytic marker) in red and FOXG1 in gray. DAPI in blue. Scale bar = 10 µm. **O** Quantifications showed that FOXG1 overexpression nearly abolished astrocytic differentiation of DG aNSPCs while the SEZ aNSPCs were unaffected compared to the CAG-GFP control cells. *n* = 3 biological replicates/group. Data represented as mean ± SEM; Welch’s *t*-test was used to determine significance if not indicated otherwise; *p < 0.05, **p < 0.01 and ***p < 0.001.

We utilized a well-established in vitro system of aNSPCs to analyze the dosage dependent effect of FOXG1 on self-renewal, proliferation, viability and differentiation of aNSPCs. In brief, we microdissected the DG and SEZ from the brains of young adult wildtype mice (P56) [31] and isolated the aNSPCs according to published protocols [32, 45, 46], which were modified by the use of the MACS neural tissue dissociation kit. To increase FOXG1 dosage, aNSPCs were transduced with an MML-retrovirus bi-cistronically encoding for murine FOXG1 and GFP—CAG-FOXG1-IRES-GFP (Fig. 2A). Control cells were transduced with an MML-retrovirus encoding for GFP-only (CAG-GFP). Average transduction efficiencies of both retroviruses were around 60% (Fig. 2B). GFP+ cells in the CAG-FOXG1-IRES-GFP transduced cultures showed an at least 3-fold higher FOXG1 immunoreactivity (Fig. 2C, D). Western blot analysis showed an average 3-fold increase in FOXG1 protein upon overexpression via the CAG-FOXG1-IRES-GFP retrovirus in all samples (Fig. 2E, F).

A single cell neurosphere assay was performed to assess the effect of FOXG1 dosage on self-renewal (Fig. 2G). Single cells were seeded into Mini-wells; 7 days later the number of formed neurospheres as a proxy for self-renewal was determined. Overexpression of FOXG1 in DG aNSPCs led to a significant decrease in self-renewal compared to the GFP-only control (Fig. 2H). In contrast, self-renewal of SEZ-derived aNSPCs was not affected by FOXG1 overexpression (Fig. 2H). For further analysis of the impact of FOXG1 on proliferation, we added the Thymidine analogue 5-Bromo-2´-Deoxyuridine (BrdU) to the cell culture medium for 3 h prior to fixation to label dividing cells (Fig. 2I). We observed a significant decrease in BrdU incorporation in FOXG1-overexpressing DG aNSPCs compared to control DG aNSPCs, indicating that FOXG1 overexpression impaired their proliferation (Fig. 2J). In contrast to DG aNSPCs and in line with the results of the self-renewal assay, proliferation of SEZ aNSPCs was unaffected by the FOXG1 overexpression. To analyze the effect of FOXG1 overexpression on viability of aNSPCs we determined the ratio of dead and living cells via Trypan Blue incorporation. While FOXG1 overexpression decreased viability of DG aNSPCs, increased FOXG1 levels did not affect the viability of SEZ aNSPCs. (Fig. 2K). We next examined the ability of FOXG1-overexpressing DG and SEZ aNSPCs to differentiate into neurons and astrocytes. Retrovirally transduced aNSPCs from DG and SEZ were differentiated for 6 days and subsequently analyzed for neuronal and astrocytic differentiation via ICC against the neuronal marker DCX and the astrocytic marker GFAP (Fig. 2L, N). Overexpression of FOXG1 almost completely abolished neuronal and astrocytic differentiation in DG aNSPCs while the differentiation of SEZ aNSPCs was unaffected by FOXG1 overexpression (Fig. 2M, O). Collectively, these in vitro analyses suggested a differential sensitivity to FOXG1 dosage of DG aNSPCs and SEZ aNSPCs.

### Adult hippocampal neurogenesis and neurogenesis in the adult SEZ/OB system show differential vulnerability to FOXG1 dosage in vivo

**Fig. 3:**
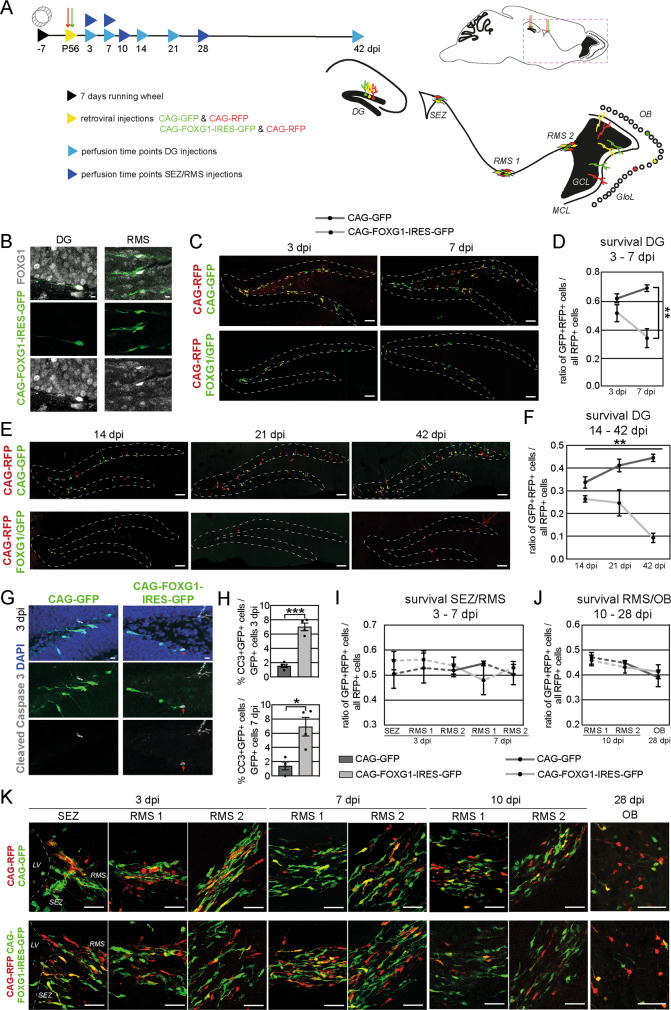
Impact of FOXG1 dosage on survival of adult-born cells in vivo. **A** Experimental scheme of retroviral injection paradigm used in vivo. After 1 week of access to running wheels adult wildtype mice were stereotactically injected either with CAG-GFP and CAG-RFP or CAG-FOXG1-IRES-GFP and CAG-RFP retroviruses into the DG or the SEZ/RMS. Animals were sacrificed either 3 dpi, 7 dpi, 14 dpi, 21 dpi and 42 dpi (DG injections) or 3 dpi, 7 dpi, 10 dpi and 28 dpi (SEZ/RMS injections). For quantifications within the RMS the two indicated positions were used. **B** Immunohistochemistry with antibodies against GFP (green) and FOXG1 (gray) in the DG and RMS verified the overexpression of FOXG1 in the CAG-FOXG1-IRES-GFP targeted cells. Scale bar = 10 µm. **C** Confocal tile scan images of retrovirus transduced adult-born control and FOXG1-overexpressing cells in the DG 3 dpi and 7 dpi. Scale bar = 100 µm. **D** The ratio of CAG-FOXG1-IRES-GFP/CAG-RFP double transduced cells (yellow cells) over all CAG-RFP transduced cells (all red cells) was significantly reduced compared to the ratio of CAG-GFP/CAG-RFP double transduced cells over all CAG-RFP transduced cells between 3 dpi and 7 dpi in the DG indicating rapid increase in cell death due to the overexpression of FOXG1. 100–200 cells analyzed/animal; *n* = 4/group. **E** Confocal tile scan images of retrovirus transduced adult-born control and FOXG1-overexpressing cells in the DG 14 dpi, 21 dpi and 42 dpi. Scale bar = 100 µm. **F** The ratio of CAG-FOXG1-IRES-GFP/CAG-RFP double transduced cells (yellow cells) over all CAG-RFP transduced cells (all red cells) was significantly reduced compared to the ratio of CAG-GFP/CAG-RFP double transduced cells over all CAG-RFP transduced cells between 14 dpi and 42 dpi in the DG indicating a further increase in cell death at later stages due to the overexpression of FOXG1. Two-Way-Anova was used to determine significance. 50–200 cells analyzed/animal; *n* = 3/group. **G** Confocal images of retrovirus transduced adult-born control and FOXG1-overexpressing cells in the DG 3 dpi. Immunohistochemistry for cleaved Caspase 3 in gray. Scale bar = 10 µm. **H** Quantifications of the apoptosis marker cleaved Caspase 3 (CC3) within the retrovirally labeled cells determined an increased ratio of CC3 + GFP +/GFP+ cells at 3 dpi and 7 dpi due to the overexpression of FOXG1 in the DG. 50–200 cells analyzed/animal; *n* = 4/group. **I**, **J** The ratio of CAG-FOXG1-IRES-GFP/CAG-RFP double transduced cells (yellow cells) over all CAG-RFP transduced cells (all red cells) was not altered compared to the ratio of CAG-GFP/CAG-RFP double transduced cells over all CAG-RFP transduced cells between 3 dpi and 7 dpi or 10 dpi and 28 dpi in the SEZ/RMS/OB. The survival of adult SEZ born cells was not altered by the overexpression of FOXG1. Solid line between time-points, dashed line between regions of the same time-point. 50–200 cells analyzed/animal; *n* = 3–4/group. **K** Confocal images of retrovirus transduced adult-born control and FOXG1-overexpressing cells in the SEZ, RMS and OB 3, dpi, 7, dpi, 10 dpi and 28 dpi (LV lateral ventricle). Scale bar = 100 µm. Data represented as mean ± SEM; Welch’s *t*-test was used to determine significance if not indicated otherwise; *p < 0.05, **p < 0.01 and ***p < 0.001.

We next sought to compare the effects of increased FOXG1 expression on neurogenesis in the DG and the SEZ/OB system in vivo. To specifically evaluate the cell-autonomous effects of increased FOXG1-levels on in vivo neurogenesis, we used MML-retroviruses to specifically target FOXG1 to fast-dividing progenitor cells without altering FOXG1 levels in surrounding cells (i.e., the neurogenic niche). Mice had access to running wheels for 7 days prior retroviral injections to increase proliferation and therefore the number of retrovirally targeted cells [47, 48]. The CAG-FOXG1-IRES-GFP MML-retrovirus was stereotactically injected into the DG and the SEZ/RMS of young adult wildtype mice (P56) (Fig. 3A). Mice injected with the CAG-GFP MML-retrovirus served as controls. Both cohorts were co-injected with an MML-retrovirus encoding for RFP as an internal control (CAG-RFP). GFP-expression was used to trace the retrovirally labeled newborn cells. Immunohistochemistry validated the overexpression of FOXG1 in the retrovirally targeted cells in the DG and RMS (Fig. 3B) and an average 3-fold increase in FOXG1 fluorescent signal intensity was determined (Fig. S1F). Animals were analyzed 3 days post injections (dpi), and 7 dpi (Fig. 3C). To assess the dosage effect of FOXG1 on early newborn cell survival, we determined the ratio of double-positive cells (GFP+ and RFP+) to all RFP+ cells in the DG. In the FOXG1 overexpression group, this ratio dropped from 0.69 on 3 dpi to 0.34 on 7 dpi, indicating a severe survival defect of the FOXG1-overexpressing adult-born cells in the DG (Fig. 3D). Immunohistochemistry for cleaved Caspase 3 (CC3), a critical protein involved in the execution of apoptosis, confirmed the survival defect as significantly more newborn DG cells overexpressing FOXG1 were positive for CC3 at 3 and 7 dpi compared to control cells (Fig. 3G, H). To determine whether FOXG1 overexpression also affected survival of newborn cells after day 7, we injected additional cohorts with CAG-FOXG1-IRES-GFP or the control CAG-GFP virus together with the CAG-RFP virus and analyzed animals on 14, 21 and 42 dpi. Again, we observed a progressive decline in the ratio of GFP + RFP+ cells/all RFP+ cells, indicating that the FOXG1 overexpression compromised newborn cell survival also at later time points (Fig. 3E, F). In contrast to the DG neurogenic niche, the ratio of GFP + RFP + cells/all RFP+ cells in the SEZ/RMS/OB system was stable across early (3–7 dpi, Fig. 3I, K) and late timepoints (10–28 dpi, Fig. 3J, K), indicating that FOXG1 overexpression did not affect survival of newborn cells in the SEZ/RMS/OB system. Moreover, the ratio of GFP + RFP+ cells/all RFP+ cells was comparable at all timepoints in all regions of the SEZ/RMS/OB system, suggesting that FOXG1 overexpression did not cause a major impairment in migration in the SEZ/RMS/OB system (Fig. 3I–K).

**Fig. 4:**
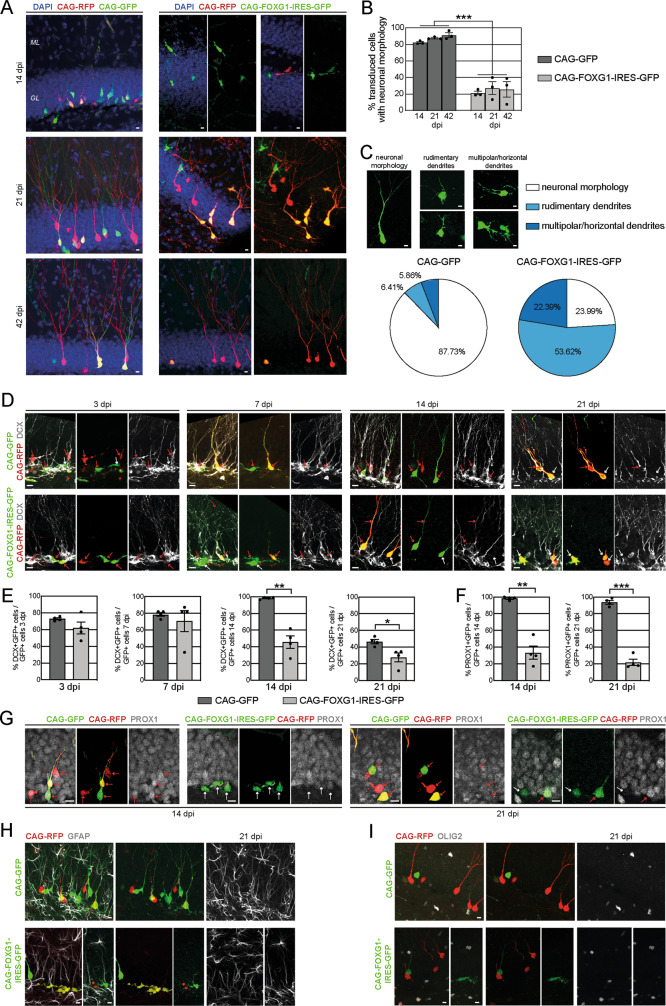
FOXG1 overexpression impairs neuronal differentiation of adult-born cells in the DG in vivo. **A** Confocal images of retrovirus transduced adult-born cells in the DG 14 dpi, 21 dpi and 42 dpi. DAPI in blue. Note that the FOXG1-overexpressing cells mostly developed an aberrant morphology while the GFP-control and the RFP-control cells developed a normal neuronal morphology defined as a cell with an apical dendrite spanning the granule layer (GL) of the DG and dendritic branching in the molecular layer (ML). Scale bar = 10 µm. **B** Quantification of the retrovirus transduced adult-born cells with neuronal morphology. Only around 20% of the FOXG1-overexpressing cells developed a neuronal morphology 14 dpi, 21 dpi and 42 dpi. 50–100 cells analyzed/animal; *n* = 3/group. **C** Confocal microscope example pictures of retrovirally labeled cells, divided in three categories: cells with neuronal morphology, cells with rudimentary dendrites and cells with multipolar/horizontal dendrites. Cumulative analyses of 14 dpi, 21 dpi and 42 dpi depicted over 50% cells with rudimentary dendrites and ca. 24% cells with multipolar/horizontal dendrites due to the overexpression of FOXG1, while only around each 6% of the GFP+ control cells showed a non-neuronal morphology. **D** Confocal images of retrovirus transduced adult-born cells in the DG 3 dpi, 7 dpi, 14 dpi and 21 dpi. Immunohistochemistry for DCX (gray) as a marker for immature neurons. Red arrows indicate DCX positive cells and white arrows indicate DCX negative cells. Scale bar = 10 µm. **E** Quantitative analysis of the neuronal differentiation with DCX as a marker for immature neurons showed no difference at 3 dpi and 7 dpi between GFP + control and GFP + FOXG1-overexpressing cells, but a significant reduction of DCX+ cells within the FOXG1-overexpressing cells (CAG-FOXG1-IRES-GFP) compared to the control group (CAG-GFP) was detected at 14 dpi and 21 dpi. 50–100 cells analyzed/animal; *n* = 4/group. **F** Quantitative analysis of the neuronal differentiation with PROX1 as a marker for DG granule neurons showed a significant reduction within the FOXG1-overexpressing cells (CAG-FOXG1-IRES-GFP) 14 dpi and 21 dpi compared to the control group (CAG-GFP). 50–100 cells analyzed/animal; *n* = 4/group. **G** Confocal images of retrovirus transduced adult-born cells in the DG 14 dpi and 21 dpi. Immunohistochemistry for PROX1 (gray) as a marker for DG neurons. Red arrows indicate PROX1 positive cells and white arrows indicate PROX1 negative cells. Scale bar = 10 µm. **H** Confocal images of retrovirus transduced adult-born cells in the DG 21 dpi. Immunohistochemistry for GFAP (gray) as a marker for astrocytes. Scale bar = 10 µm. **I** Confocal images of retrovirus transduced adult-born cells in the DG 21 dpi. Immunohistochemistry for OLIG2 (gray) as pan-oligodendrocyte marker. Scale bar = 10 µm. Data represented as mean ± SEM; Welch’s *t*-test was used to determine significance if not indicated otherwise; *p < 0.05, **p < 0.01 and ***p < 0.001.

Inspection of the surviving FOXG1-overexpressing DG cells revealed an aberrant morphology (Fig. 4A). While control cells (GFP+ cells in the control animals or RFP-only cells in the CAG-FOXG1-IRES-GFP injected animals) featured the stereotypic dentate granule neuron morphology with an apical dendrite spanning the GCL of the DG and branching dendrites in the molecular layer (Fig. 4A), around 80% of the FOXG1-overexpressing cells in the DG lacked this stereotypic morphology (Fig. 4B). We further divided the newborn cells morphologically in cells with neuronal morphology, cells with only rudimentary dendritic development and cells with multipolar/horizontal dendrites (Fig. 4 C). Cumulative analysis from 14 to 42 dpi showed that more than 50% of the FOXG1-overexpressing cells showed a morphology with just rudimentary dendrites, around 22% had multipolar/horizontal dendrites and only around 24% showed the prototypic dentate granule neuronal morphology (Fig. 4C). In contrast, GFP-positive control cells developed in up to 90% the expected neuronal morphology and only around 12% showed a multipolar/horizontal or rudimentary dendritic morphology. We next performed phenotyping of retrovirally transduced cells using DCX as a marker for immature neurons and PROX1 (Prospero Homeobox Protein 1) as a dentate granule neuron specific marker (Fig. 4D–G). At 3 and 7 dpi 80% of all GFP-positive control cells were DCX+ and 14 dpi next to all GFP-positive control cells were DCX+ (Fig. 4E). Consistent with previous reports [49], this fraction dropped to 50% at 21 dpi as a sign of further maturation (Fig. 4E). Moreover, GFP-positive control cells were almost invariably PROX1+ at 14 dpi and 21 dpi indicating that cells had adopted a dentate granule neuron identity (Fig. 4F, G). At 3 dpi and 7 dpi no significant differences were detectable in the fraction of GFP + DCX + cells between control and FOXG1-overexpressing cells (Fig. 4D, E). At later time-points, however, only one half (14 dpi) and one third (21 dpi) of the surviving FOXG1-overexpressing cells were positive for DCX (Fig. 4D, E). In addition, only 30% of the FOXG1-overexpressing cells expressed the DG specific neuronal marker PROX1 at 14 dpi and this number decreased further to about 20% at 21 dpi (Fig. 4F, G).

Since most of the surviving FOXG1-overexpressing cells in the DG did not express markers consistent with a neuronal fate, we tested whether they had instead differentiated into glia. Cells were, however, negative for GFAP as an astrocytic marker and OLIG2 (Oligodendrocyte Transcription Factor 2) as a pan-oligodendrocytic marker (Fig. 4H, I) indicating that FOXG1 overexpression did not promote differentiation of adult-born cells in the DG into glia.

**Fig. 5:**
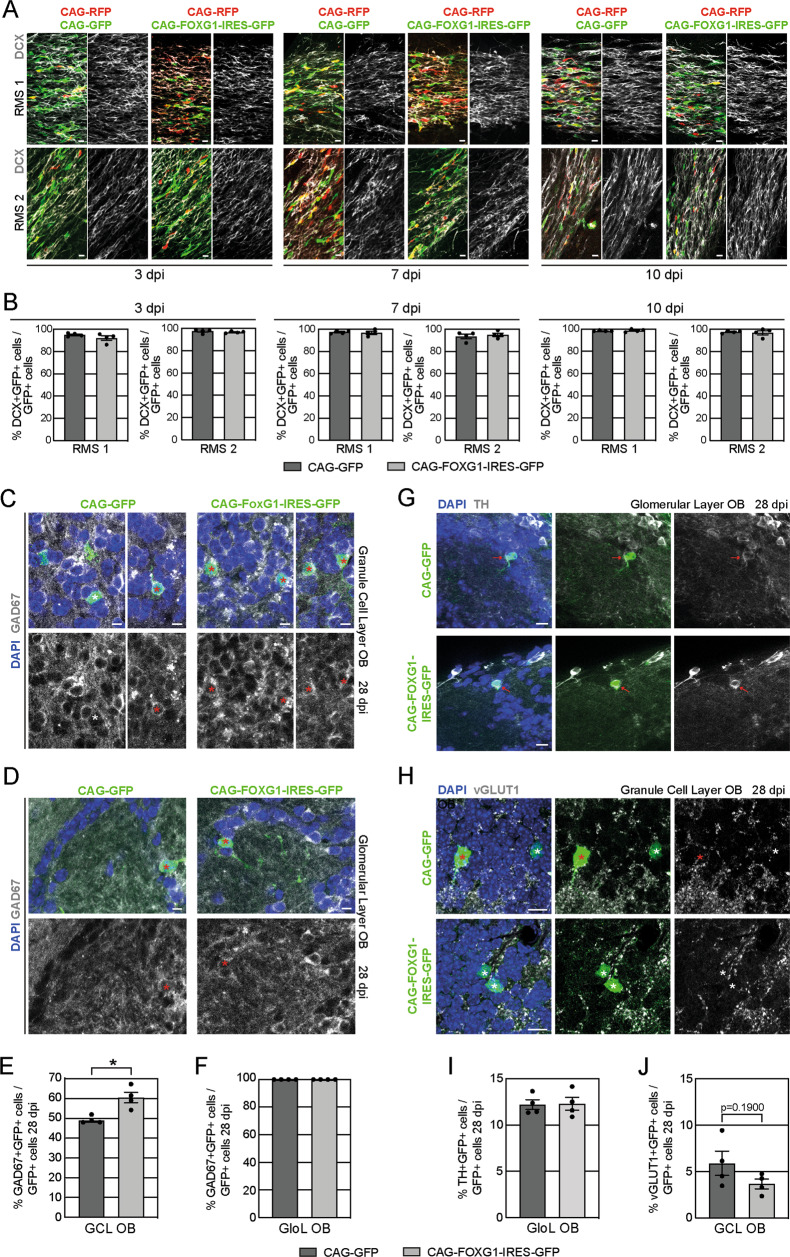
FOXG1 overexpression does not impair adult-born cells in the SEZ/RMS/OB in vivo. **A** Confocal images of retrovirus transduced adult-born cells of the SEZ within two positions of the RMS (RMS 1 and RMS 2) at 3 dpi, 7 dpi and 10 dpi. Immunohistochemistry for DCX (gray) as a marker for migrating neuroblasts. Scale bar = 10 µm. **B** Quantitative analysis of the DCX+ neuroblasts, migrating through the RMS showed no differences between the control cells (CAG-GFP transduced, CAG-RFP transduced) or the FOXG1-overexpressing cells (CAG-FOXG1-IRES-GFP) at 3 dpi, 7 dpi or 10 dpi. Nearly all transduced cells were positive for DCX within the RMS. 100–200 cells analyzed/animal; *n* = 4/group. Confocal images of retrovirus transduced adult-born cells of the SEZ within the GCL (**C**) and the GloL (**D**) of the OB 28 dpi. Immunohistochemistry for GAD67 (gray) as a marker for GABAergic neurons. DAPI in blue. White asterisks indicate GAD67 negative cells and red asterisks indicate GAD67 positive cells. Scale bar = 10 µm. **E**, **F** Quantitative analysis of the percentage of GAD67 + GABAergic neurons within the retroviral transduced cells in the GCL and GloL of the OB 28 dpi. Overexpression of FOXG1 led to a significant increase in the fraction of newborn GAD67+ cells within the GCL of the OB 28 dpi. 50–100 cells analyzed/animal; *n* = 4/group. **G** Confocal images of retrovirus transduced adult-born cells of the SEZ within the GloL of the OB 28 dpi. Immunohistochemistry for TH (gray) as a marker for dopaminergic neurons. DAPI in blue. Red arrows indicate TH positive cells. Scale bar = 10 µm. **H** Confocal images of retrovirus transduced adult-born cells of the SEZ within the GCL of the OB 28 dpi. Immunohistochemistry for VGLUT1 (gray) as a marker for glutamatergic neurons. DAPI in blue. Red asterisks indicate VGLUT1 positive cells and white asterisks indicate VGLUT1 negative cells. Scale bar = 10 µm. **I** Quantitative analysis of the percentage of TH+ dopaminergic neurons within the retroviral transduced cells in the GloL of the OB 28 dpi. Overexpression of FOXG1 did not alter the number of newborn TH+ cells within the GloL of the OB 28 dpi. 100 cells analyzed/animal; *n* = 4/group. **J** Quantitative analysis of the percentage of VGLUT1+ glutamatergic neurons within the retroviral transduced cells in the GCL of the OB 28 dpi. Overexpression of FOXG1 did led to a slight but not significant decrease of newborn VGLUT1+ cells within the GCL of the OB 28 dpi. 100 cells analyzed/animal; *n* = 4/group. Data represented as mean ± SEM; Welch’s *t*-test was used to determine significance if not indicated otherwise; *p < 0.05, **p < 0.01 and ***p < 0.001.

Adult SEZ born cells migrate as DCX+ neuroblasts via the RMS to the OB where they differentiate into mature neurons. Virtually all GFP-positive control cells and FOXG1-overexpressing cells were positive for DCX at 3 dpi, 7 dpi and 10 dpi while migrating through the RMS (Fig. 5A, B). FOXG1-overexpressing cells had the prototypic bipolar shape of migrating neurons in the RMS and were morphologically indistinguishable from the RFP+ internal control cells (Fig. 5A). At 28 dpi 50% of the GFP+ control cells in the GCL of the OB expressed the GABAergic marker GAD67 (Fig. 5C, E). The percentage of FOXG1-overexpressing neurons in the GCL of the OB expressing GAD67 was slightly increased to 60% (Fig. 5C, E). Adult-born GFP-positive control and FOXG1-overexpressing cells located in the GloL were positive for GAD67 (Fig. 5D, F). Although, most of the adult SEZ-born OB neurons are GABAergic, a small percentage of glutamatergic granule layer neurons and dopaminergic periglomerular neurons are generated as well [50–52]. Therefore, we used TH (Tyrosine Hydroxylase) as a marker for dopaminergic neurons in the GloL of the OB and VGLUT1 (Vesicular Glutamate Transporter 1) as marker for glutamatergic neurons in the GCL of the OB (Fig. 5G, H). The fraction of adult-born dopaminergic periglomerular neurons in the GloL was not altered by the overexpression of FOXG1 (Fig. 5I). However, we detected a slight but not significant decrease in the fraction of adult-born glutamatergic neurons following overexpression of FOXG1 in the GCL of the OB (Fig. 5J). We further determined the morphology of the adult-born granule OB neurons to assess any further effects of FOXG1 gain-of-function at 28 dpi. Therefore, we divided the retrovirally labeled cells according to their position in deep layer and superficial layer OB neurons (Fig. S2A) and reconstructed them via the Fiji ImageJ plugin Simple Neurite Tracer (Fig. S2B) [53]. No significant changes in total dendritic length, number of branch points or dendrite termini between GFP + FOXG1-overexpressing and GFP+ control cells of the deep or superficial OB layers were detected (Fig. S2C). Also, Sholl analyses did not reveal any alterations in cell complexity due to the overexpression of FOXG1 at 28 dpi (Fig. S2D). As a proxy for functionality and maturation, we analyzed the number of spines per dendritic length and analyzed the expression of the immediate early gene *cfos* as a marker for neuronal activity and functional incorporation of adult-born neurons into the neuronal circuitry (Fig. S2E–H) [54–56]. Neither the number of spines nor the number of GFP + cFOS+ cells was altered by FOXG1 overexpression compared to control cells at 28 dpi (Fig. S2F, H), indicating that FOXG1 gain-of-function did not impair functionality of adult-born OB neurons.

Collectively, these data indicate that increased FOXG1 expression—in contrast to its detrimental effects on the generation of glutamatergic dentate granule neurons—does not affect the generation of GABAergic neurons in the SEZ/OB system.

### RNA-sequencing reveals an apoptotic signaling program after FOXG1 overexpression only in DG aNSPCs

**Fig. 6:**
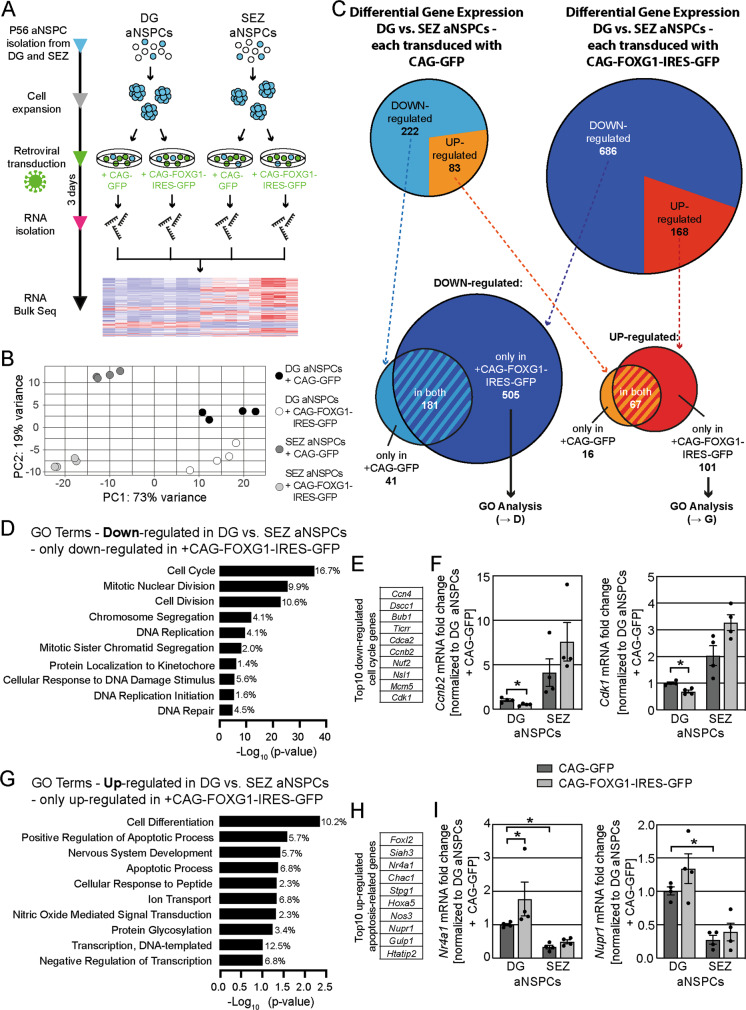
FOXG1 overexpression induces an apoptotic signaling program only in DG aNSPCs. **A** Experimental scheme of sample preparation for Bulk RNA-seq. DG and SEZ aNSPCs were isolated from adult mice (P56, *n* = 4), expanded and transduced with a GFP-encoding control (CAG-GFP) or a FOXG1-overexpressing (CAG-FOXG1-IRES-GFP) retrovirus. Cells were harvested 3 days after retroviral transduction, total RNA isolated and processed for Bulk RNA-seq. RNA-seq was performed on four independent samples per group. **B** Principial component analysis distinguished DG from SEZ aNSPC samples (Principal component 1 PC1) and CAG-GFP from CAG-FOXG1-IRES-GFP transduced samples (PC2). **C** Pie charts of the differentially expressed genes (log2 fold change >1.5 or < −1.5; *p* < 0.01) either DG vs. SEZ aNSPCs each transduced with CAG-GFP (light blue = number of downregulated genes, orange = number of upregulated genes) or DG vs. SEZ aNSPCs each transduced with CAG-FOXG1-IRES-GFP (dark blue = number of downregulated genes, red = number of upregulated genes). Venn diagram of the number of genes that were downregulated in DG vs. SEZ aNSPCs only if transduced with the CAG-GFP control retrovirus (light blue), or only if transduced with the CAG-FOXG1-IRES-GFP overexpression retrovirus (dark blue), or in both (light and dark blue striped). Venn diagram of the number of genes that were upregulated in DG vs. SEZ aNSPCs only if transduced with the CAG-GFP control retrovirus (orange), or only if transduced with the CAG-FOXG1-IRES-GFP overexpression retrovirus (red), or in both (orange and red striped). The genes, which were only downregulated or upregulated in DG vs. SEZ aNSPCs after FOXG1 overexpression were used for Gene Ontology (GO) analyses. **D** GO analysis of the 505 downregulated genes in DG vs. SEZ aNSPCs downregulated only after FOXG1 overexpression (the smaller the *p*-value, the more significantly downregulated; percentage of differentially regulated/total genes in the term). **E** The top 10 downregulated cell-cycle-related genes from GO analysis (according to log2 fold change). **F** Quantitative PCR analysis (qRT-PCR) of gene expression for the cell-cycle-related genes *Ccnb2* and *Cdk1*. *n* = 4 biological replicates/group. **G** GO analysis of the 101 upregulated genes in DG vs. SEZ aNSPCs only upregulated after FOXG1 overexpression (the smaller the *p*-value, the more significantly upregulated; percentage of differentially regulated/total genes in the term). **H** The top 10 upregulated apoptosis-related genes from GO analysis (according to log2 fold change). **I** Quantitative PCR analysis (qRT-PCR) of gene expression for the apoptosis-related genes *Nr4a1* and *Nupr1*. *n* = 4 biological replicates/group. Data represented as mean ± SEM; Welch’s *t*-test was used to determine significance if not indicated otherwise; *p < 0.05, **p < 0.01 and ***p < 0.001.

To uncover candidate pathways underlying the differential vulnerability of the DG and SEZ/OB neurogenic system, we compared the transcriptomic changes induced by FOXG1 overexpression between DG and SEZ aNSPCs. DG and SEZ aNSPCs were retrovirally transduced with the CAG-FOXG1-IRES-GFP retrovirus or the GFP-expressing control retrovirus (CAG-GFP) (Fig. 6A). Three days after retroviral transduction, cells were harvested, and analyzed by RNA sequencing (RNA-seq). In principal component analysis (PCA) the four samples showed distinct separation based on PC1 and PC2, which covered >90% of the variance (Fig. 6B). PC1 distinguished between DG and SEZ aNSPCs and PC2 between control and FOXG1 overexpression. DG aNSPCs and SEZ aNSPCs had distinct transcriptomic signatures with 222 genes were downregulated and 83 upregulated in the comparison of GFP-control retrovirus transduced DG aNSPCs and SEZ aNSPCs (Figs. 6C, S3A, D, Table S1). Moreover, the different aNSPC cultures showed distinct pathway activities as revealed by KEGG Pathways analysis of differentially expressed genes using DAVID [39, 40] (Fig. S3B, Table S1). In the comparison of FOXG1-overexpressing DG aNSPCs and SEZ aNSPCs 686 genes were downregulated and 168 genes were upregulated (Figs. 6C, S3C, D, Table S1). We compared the lists of differentially downregulated or upregulated genes to distinguish those genes, whose differential expression was most likely reflecting the distinct regional identity of the samples, from genes, whose differential expression between DG aNSPCs and SEZ aNSPCs was the result of the FOXG1 overexpression (Fig. 6C, Table S1). In a next step, we performed GO-term analyses of the identified FOXG1-dependent downregulated and upregulated genes using DAVID (Fig. 6D, G, Table S1) [39, 40]. Consistent with the DG specific phenotypes of decreased proliferation, increased cell death, and impaired differentiation following FOXG1 overexpression, the most prominent downregulated GO-terms were related to cell cycle and cell proliferation (Fig. 6D), whereas the most prominent upregulated GO-terms were associated with nervous system development and apoptotic processes (Fig. 6G). The top 10 cell-cycle-related downregulated genes encoded for cyclins, proteins involved in cell survival, DNA replication and regulation, mitotic spindle checkpoints, chromosome structure and segregation, microtubule binding and regulation of mitosis (Fig. 6E). We confirmed the downregulation of the cyclin *Ccnb2* (Cyclin B2) and the regulator of mitosis *Cdk1* (Cyclin Dependent Kinase 1) in DG aNSPCs overexpressing FOXG1 by qRT-PCR analysis (Fig. 6F). The top 10 apoptosis-related upregulated genes encoded proteins involved in induction and promotion of apoptosis, cell cycle arrest, transcriptional regulation upon cellular stress and apoptotic engulfment (Fig. 6H). We confirmed the upregulation of the inducers of apoptosis *Nr4a1* (Nuclear receptor 4 a1) and *Nupr1* (Nuclear protein 1) upon FOXG1 overexpression in DG aNSPCs by qRT-PCR analysis (Fig. 6I).

**Fig. 7:**
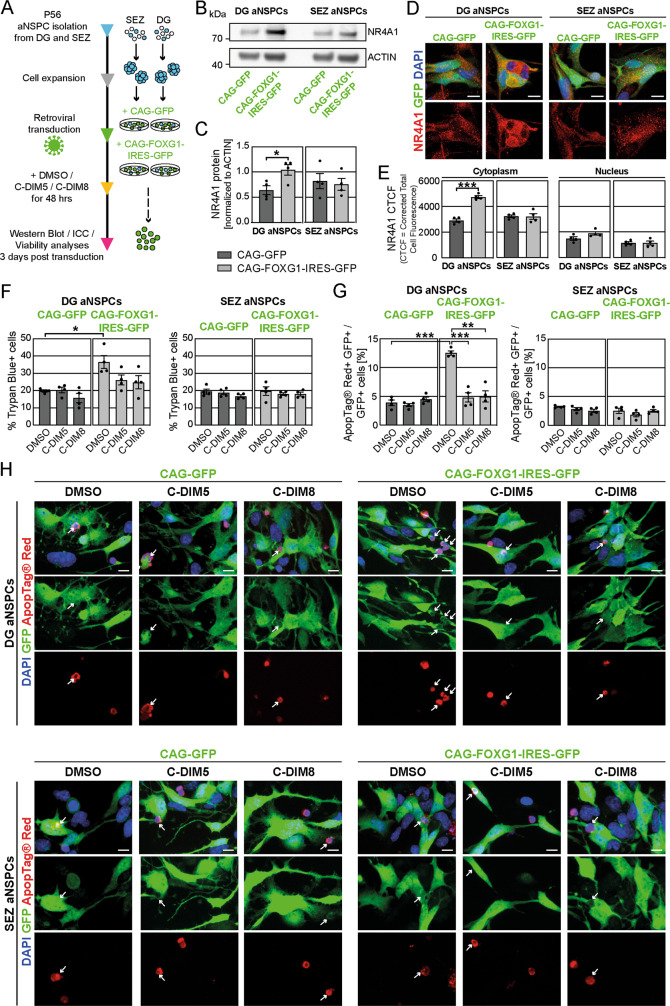
Pharmacological interference with NR4A1 function rescues the apoptosis phenotype of DG aNSPCs upon FOXG1 overexpression in vitro. **A** Experimental paradigm to analyze FOXG1 dosage in aNSPCs in vitro. aNSPCs were isolated from the DG and the SEZ of wildtype mice at P56. Cells were expanded and transduced with either a GFP-encoding control retrovirus or a FOXG1-IRES-GFP-encoding retrovirus. Cells were treated as indicated and used for western blot analysis, immunocytochemistry, and viability analyses. **B** Western blot analysis of protein lysates of DG and SEZ aNSPCs transduced with either CAG-GFP control or the FOXG1 overexpression (CAG-FOXG1-IRES-GFP) retrovirus. An antibody against NR4A1 was applied. ACTIN was used as endogenous loading control. **C** For quantification, protein amounts were normalized to ACTIN. NR4A1 protein was significantly increased in DG aNSPCs upon FOXG1 overexpression. *n* = 4 biological replicates/group. **D** ApoTome images of aNSPCs from DG and SEZ transduced with either a CAG-GFP control retrovirus or a CAG-FOXG1-IRES-GFP retrovirus. Immunocytochemistry for NR4A1 in red. DAPI in blue. Scale bar = 10 µm. **E** Corrected Total Cell Fluorescence-based (CTCF) quantifications revealed that FOXG1 overexpression significantly increased immunoreactivity for NR4A1 only in the cytoplasm of DG aNSPCs. *n* = 4 biological replicates/group. **F** Viability assay via Trypan Blue incorporation showed increased cell death in DG aNSPCs due to FOXG1 overexpression, which was diminished by C-DIM5 (15 µM for 48 h) or C-DIM8 (15 µM for 48 h) treatment. The viability of SEZ aNSPCs was not altered by FOXG1 overexpression or C-DIM5 or C-DIM8 treatment. *n* = 4 biological replicates/group. **G** Apoptosis analysis via the ApopTag^®^ Red In Situ Detection Kit. Quantifications of ApopTag^®^ Red-positive cells within the GFP-positive retrovirally transduced cells depicted a significant increase in apoptotic cells after FOXG1 overexpression in DG aNSPCs, which was prevented via C-DIM5 (15 µM for 48 h) or C-DIM8 (15 µM for 48 h) treatment. The percentage of apoptotic SEZ aNSPCs was not altered by FOXG1 overexpression or C-DIM5 or C-DIM8 treatment. *n* = 4 biological replicates/group. **H** ApoTome images of aNSPCs from DG and SEZ transduced with either a CAG-GFP control retrovirus or a CAG-FOXG1-IRES-GFP retrovirus and treated either with DMSO (vehicle control) or C-DIM5 (15 µM for 48 h) or C-DIM8 (15 µM for 48 h). The ApopTag^®^ Red In Situ Detection Kit was used to detect apoptotic cells, depicted in red. DAPI in blue. Scale bar = 10 µm. Data represented as mean ± SEM; Welch’s *t*-test was used to determine significance if not indicated otherwise; *p < 0.05, **p < 0.01 and ***p < 0.001.

Together with the functional data the transcriptomic analysis suggested that increased FOXG1 expression induced an apoptotic program specifically in DG aNSPCs. One of the most upregulated genes in the comparison of DG aNSPCs vs. SEZ aNSPCs due to FOXG1 overexpression, which has been shown to be involved in the induction of apoptosis, was *Nr4a1*. NR4A1 is an orphan nuclear receptor with context-dependent regulatory functions in proliferation, differentiation and apoptosis in various tissues [57]. Nuclear NR4A1 can transcriptionally regulate the expression of both apoptotic and anti-apoptotic programs. NR4A1 can translocate to the cytoplasm and bind to BCL2 (B-cell lymphoma 2), thereby targeting BCL2 to mitochondria, which promotes cytochrome c release and apoptosis [58–60]. Western blot analysis confirmed a significant increase of NR4A1 protein levels in DG aNSPCs but not in SEZ aNSPCs upon FOXG1 overexpression (Fig. 7B, C). To further investigate the expression of NR4A1, we performed ICC and analyzed the fluorescence signal of NR4A1 relative to cell area calculating the CTCF within the cytoplasm and within the nucleus (Fig. 7D). Interestingly, the fluorescence signal of NR4A1 was significantly upregulated in the cytoplasm of DG aNSPCs but not in SEZ aNSPCs upon FOXG1 overexpression (Fig. 7E). We could not detect any differences in nuclear NR4A1 expression between DG and SEZ aNSPCs with or without FOXG1 overexpression (Fig. 7E). To investigate whether increased NR4A1 activity contributes to the apoptosis phenotype in DG aNSPCs after FOXG1 overexpression, we treated cultures with the C-DIM8 compound (DIM-C-pPhOH, Selleck Chemicals) or the C-DIM5 compound (DIM-C-pPhOCH3, Selleck Chemicals). C-DIM8 has been shown to act as a classical antagonist that reduces NR4A1-dependent transcriptional activity [61]; C-DIM5 promotes nuclear retention of NR4A1 and reduces cytoplasmic levels of NR4A1 [62, 63]. Testing different doses of the compounds (0–100 µM) we found that DG and SEZ aNSPCs could be treated for 48 h with 15 µM C-DIM5 or 15 µM C-DIM8 without significant induction of cell death (Fig. S4A). Next, DG and SEZ aNSPCs, transduced with the CAG-GFP control and CAG-FOXG1-IRES-GFP overexpression retroviruses, were treated either with the vehicle control (DMSO), 15 µM C-DIM5 or 15 µM C-DIM8 for 48 h. Notably, analyses of the cytoplasmic and nuclear NR4A1 fluorescence signal (Fig. S4B, C) showed that both compounds significantly reduced the cytoplasmic NR4A1 signal. In addition, C-DIM5 treatment significantly increased the nuclear NR4A1 signal. To determine cell viability, we first analyzed the number of Trypan Blue positive cells in DG and SEZ aNSPCs under control (CAG-GFP) and FOXG1-overexpression (CAG-FOXG1-IRES-GFP) conditions treated either with DMSO (vehicle control), or C-DIM5 (15 µM) or C-DIM8 (15 µM) for 48 h (Fig. 7F). As expected, DMSO treated DG aNSPCs with FOXG1 overexpression showed significantly increased numbers of Trypan Blue+ cells compared to DMSO treated DG aNSPCs control cells. FOXG1-overexpressing DG aNSPCs showed a trend towards increased cell viability in the C-DIM5 and C-DIM8 treatment groups. C-DIM5 and C-DIM8 treatment did not alter cell viability of control transduced DG aNSPCs. To more specifically analyze the impact of the NR4A1 inhibitors on apoptosis in FOXG1-overexpressing aNSPCs, we analyzed cells using the ApopTag^®^ Red in situ Apoptosis Kit (Fig. 7G, H). FOXG1-overexpressing DG aNSPCs showed a significantly higher fraction of GFP+ cells positive for ApopTag^®^ Red compared to control DG aNSPCs. In the presence of the C-DIM5 or C-DIM8, the fraction of apoptotic cells amongst FOXG1-overexpressing DG aNSPCs dropped to control levels. Notably, C-DIM5 or C-DIM8 treatment did not significantly affect apoptosis levels amongst control transduced DG aNSPCs. Moreover, neither FOXG1 overexpression nor treatment with NR4A1 inhibitors significantly altered cell viability and apoptosis of SEZ aNSPCs (Fig. 7F, G). Collectively, these data indicate that a NR4A1-dependent apoptotic pathway contributes to the differential vulnerability of DG vs. SEZ aNSPCs to increased FOXG1 expression.

## Discussion

Previous work demonstrated that FOXG1 is required for dentate granule neuron and DG development and that the efficient generation of dentate granule neurons is highly sensitive to decreased FOXG1 dosage [64, 65]. Here, we report that the generation of dentate granule neurons in the adult murine hippocampus is highly vulnerable to increased FOXG1 dosage. Abnormally high levels of FOXG1 led to pronounced death of progenitor cells and developing neurons, and was associated with impaired aNSPC proliferation, neuronal differentiation, and expression of the dentate granule neuron phenotype.

Given the pronounced cell death, the direct impact of FOXG1 overexpression on proliferation and differentiation pathways in the adult hippocampal neurogenic lineage is difficult to judge. Transcriptomic analyses, however, revealed a downregulation of cell-cycle related genes in FOXG1-overexpressing DG aNSPCs, suggesting that FOXG1 may have a direct effect on proliferation. FOXG1 represses dentate granule cell fate in telencephalic progenitors during mouse embryonic development [66]. In the present study, sustained high levels of FOXG1 reduced the expression of the dentate granule neuron specific transcription factor PROX1 and inhibited the development of the dentate granule neuron specific dendritic arbor, indicating that FOXG1-overexpressing cells failed to adopt or to maintain a dentate granule neuron identity, which in turn impeded on their functional integration and survival [43, 67].

In contrast to neurogenesis in the adult DG, neurogenesis in the SEZ/RMS/OB system was unaffected by FOXG1 overexpression with regard to the parameters cell death, region-specific differentiation (except for the generation of glutamatergic neurons of the GCL), and morphological and histological indicators of functional integration. It has, however, to be noted that we cannot exclude that FOXG1 overexpression in newly generated OB neurons may have an impact on olfactory information processing. The reason for the differential sensitivity of DG and SEZ/RMS/OB neurogenesis remains to be determined. NSPCs in the adult SEZ/OB system mainly produce GABAergic interneurons [68, 69], which in light of the GABAergic fate promoting function of FOXG1 [70, 71] may render the SEZ/OB system more resilient to aberrant levels of FOXG1. Such hypothesis would be supported by the observation that FOXG1 overexpression appeared to impair the generation of non-GABA-expressing glutamatergic neurons, which are generated in small numbers in the adult SEZ/OB neurogenic system [50].

Our finding that DG aNSPCs but not SEZ aNSPCs respond with increased apoptosis to increased FOXG1 levels, indicate that aNSPCs of the two neurogenic systems display differential vulnerability to increased FOXG1 levels. We performed RNA-Seq analysis to identify candidate pathways conferring the DG aNSPC-specific cell death response to supraphysiological FOXG1 levels. Under identical culture conditions, aNSPCs from the DG and the SEZ showed distinct transcriptional profiles, supporting the idea that these regions harbor distinct aNSPC populations [45], that are primed to produce region-specific neuronal subtypes. Consistent with the cellular phenotype of impaired proliferation and cell death, we observed the differential expression of cell cycle and apoptosis-related genes specifically in FOXG1-overexpressing DG aNSPCs. The orphan nuclear receptor *Nr4a1* was amongst the top 3 differentially regulated apoptosis-related genes. Treatment with the pharmacological modulators of NR4A1 function C-DIM5 and C-DIM8 rescued FOXG1-dependent apoptosis of DG aNSPCs. This finding was surprising, as C-DIM5 and C-DIM8 function as inhibitor and activator of NR4A1-dependent transcription, respectively. NR4A1 controls apoptosis not only via transcription-dependent but also independent pathways, the latter involving the binding of cytoplasmic NR4A1 to BCL2 (B-cell lymphoma 2), which then targets BCL2 to mitochondria to induce cytochrome c release and apoptosis [58–60]. Interestingly, while C-DIM5 and C-DIM8 showed opposite effects on nuclear NR4A1 levels, both compounds significantly lowered cytoplasmic levels of NR4A1, indicating that NR4A1 promoted cell death of FOXG1-overexpressing DG aNSPCs primarily via the transcription-independent cytoplasmic pathway. Notably, wildtype DG, wildtype SEZ, and FOXG1-overexpressing SEZ aNSPCs expressed baseline levels of NR4A1; pharmacological manipulation of NR4A1 function in these cells, however, did not affect their viability, supporting the notion that cytoplasmic NR4A1-dependent signaling has a specific function in FOXG1-induced cell death of DG aNSPCs.

How FOXG1 regulates NR4A1 expression and cytoplasmic localization remains to be determined. Current data indicate that FOXG1 acts primarily as a transcriptional repressor, suggesting that FOXG1 may not directly target *Nr4a1* expression but may instead control the activity of upstream regulators of *Nr4a1* expression and NR4A1 localization. Future studies should also address the mechanism underlying the difference in FOXG1-dependent *Nr4a1* induction between DG and SEZ aNSPCs.

It is interesting to note that while protein levels of FOXG1 and NR4A1 were comparable between DG and SEZ aNSPCs (Figs. 2E, F; 7B–E), their mRNA levels differed between these groups (Fig. 6I; Supplemental Table S1). This observation suggests the interesting idea, that both FOXG1 and NR4A1 expression may be subject to distinct regulatory mechanisms in DG and SEZ aNSPCs.

Gene duplications of *FOXG1* have been causally linked to a neurodevelopmental syndrome (*FOXG1* syndrome) featuring intellectual disability and autistic behavior [16–20]. Recent data suggest that the cognitive and behavioral symptoms in *FOXG1*-syndrome are at least in part the consequence of altered excitability and disruption of the E/I balance in neural circuits [25, 72]. E/I imbalance [23, 24] and increased FOXG1 expression [8, 21, 25] are also hypothesized to be contributing factors in the pathogenesis of idiopathic ASD. Our findings raise the interesting possibility that differential sensitivity of glutamatergic and of GABAergic NSPCs to FOXG1 dosage may disrupt the balanced generation of excitatory and inhibitory neurons. It will therefore be interesting to determine, how FOXG1 protein levels in *FOXG1* syndrome compare to FOXG1 protein levels in this study and whether glutamatergic and GABAergic NSPCs in the developing CNS display differential sensitivity to FOXG1 dosage. It will also be important to study the sensitivity of glutamatergic and of GABAergic aNSPCs in a setting, in which FOXG1 levels are increased not only in NSPCs but also in surrounding cells. Finally, given that both, increased and decreased FOXG1 dosage cause *FOXG1* syndrome, future studies should also explore the effects of decreased FoxG1 dosage on distinct NSPC populations.

## Supplementary information

Supplementary Figure legends

Supplementary Figure S1

Supplementary Figure S2

Supplementary Figure S3

Supplementary Figure S4

Supplementary Table S1
