# Supplementary material for: Differential vulnerability of adult neurogenic niches to dosage of the neurodevelopmental-disorder linked gene Foxg1
Source: Mol Psychiatry. 2022 Mar 22;28(1):497–514. doi: 10.1038/s41380-022-01497-8 (PMC9812795; doi:10.1038/s41380-022-01497-8)
Supplement: Supplementary file 1 — Supplementary Figure legends [file 41380_2022_1497_MOESM1_ESM.docx]

**Supplementary Figure Legends**

**Supplementary Figure S1. FOXG1 antibody validation and FOXG1 intensity measurements *in vivo*. Related to Figure 1 and 3.**

**A** Breeding scheme to generate FOXG1 WT and FOXG1 KO mice to validate the applied FOXG1 antibodies. As the full KO of FOXG1 is perinatal lethal^1^, heterozygous mice with one WT allele and one allele in which Cre is targeted to the *Foxg1* locus (*Foxg1*^Cre/+)^ were mated to generate FOXG1 WT and FOXG1 KO littermates. Litters were sacrificed at embryonic day 13.5 (E13.5).

**B** Schematic coronal view of an E13.5 mouse brain; red box indicates area chosen for imaging in C and D.

**C, D** Confocal images with 25x and 63x oil objectives of FOXG1 WT (*Foxg1^+/+^*) and FOXG1 KO (*Foxg1^Cre/Cre^*) cortices at E13.5. Immunohistochemistry for the utilized FOXG1 antibodies in grey. DAPI in blue. Both applied FOXG1 antibodies showed a mainly nuclear signal in the Cortex of FOXG1 WT, while there was no distinct signal detectable in FOXG1 KO animals. Scale bar = 10 µm.

**E** FOXG1 fluorescent signal intensity measurements (mean pixel value) of SOX2+GFAP+ neural stem cells, SOX2+ progenitors, TBR2+ transient amplifying progenitors, DCX+ neuroblasts/immature neurons and Map2+ mature neurons in the adult DG and of SOX2+GFAP+ neural stem cells, SOX2+ progenitors, DCX+ neuroblasts, NEUN+ neurons and GAD67+ GABAergic neurons in the adult SEZ/RMS/OB. 20 cells analyzed / animal; n = 3.

**F** Measurement of FOXG1 fluorescent signal intensities (mean pixel value) in CAG-GFP or CAG-FOXG1-IRES-GFP transduced cells in the DG or RMS showed a 3-fold increase in FOXG1 signal after FOXG1-overexpression *in vivo*. 50 cells analyzed / animal; n = 4 / group.

**Supplementary Figure S2. Morphological analyses of adult-born olfactory bulb neurons overexpressing FOXG1 *in vivo*. Related to Figure 5.**

**A** Confocal images of retrovirus transduced adult-born cells of the SEZ within the deep and superficial layers of the OB transduced either with a GFP-encoding control or a FOXG1-overexpressing retrovirus 28 dpi. DAPI in blue. Scale bar = 100 µm.

**B** Representative reconstructions of GFP control and FOXG1-overexpressing deep layer neurons and superficial neurons of the OB at 28 dpi. Scale bar = 100 µm.

**C** Quantifications of the total dendritic length, number of dendrite branch points and number of dendrite termini of control and FOXG1-overexpressing deep layer and superficial neurons in the adult OB at 28 dpi showed no differences between control and FOXG1-overexpressing neurons. 10 cells analyzed / animal; n = 4 / group.

**D** Sholl analysis of 28 dpi deep layer and superficial OB neurons did not differ between GFP+ control and FOXG1-overexpressing neurons. 10 cells analyzed / animal; n = 4 / group.

**E** Representative confocal microscope images showing dendritic segments of adult-born GFP+ control and FOXG1-overexpressing neurons, at 28 dpi.

**F** Spine density was not altered due to FOXG1 overexpression. 10 cells analyzed / animal; n = 4 / group.

**G** Confocal images of adult-born cells of the SEZ within the granule layer of the OB transduced either with a GFP-encoding control or a FOXG1-overexpressing retrovirus 28 dpi. Immunohistochemistry for the immediate-early gene cFOS in grey. DAPI in blue. Scale bar = 100 µm.

**H** The percentage of cFOS+ cells amongst all GFP+ cells was not altered due to overexpression of FOXG1. 100 cells analyzed / animal; n = 4 / group.

Data represented as mean ± SEM; Welch’s t-test was used to determine significance if not indicated otherwise; *p<0.05, **p<0.01 and ***p<0.001.

**Supplementary Figure S3. Differentially expressed genes in DG and SEZ aNSPCs upon FOXG1 overexpression. Related to Figure 6.**

**A** Heatmap of the differentially expressed genes shown as Z-score for all replicates of each group (DG vs. SEZ aNSPCs each transduced with the CAG-GFP control retrovirus).

**B** KEGG Pathways analysis of up- and down-regulated genes in DG vs. SEZ aNSPCs transduced with the CAG-GFP control retrovirus (the smaller the p-value, the more significantly up- or down-regulated).

**C** Heatmap of the differentially expressed genes shown as Z-score for all replicates of each group (DG vs. SEZ aNSPCs each transduced with the FOXG1 overexpression retrovirus).

**D** Number of differentially expressed genes according to their log2 fold change in DG vs. SEZ aNSPCs transduced either with a GFP-encoding control (CAG-GFP) or FOXG1-overexpressing retrovirus (CAG-FOXG1-IRES-GFP).

**Supplementary Figure S4. C-DIM5/8 dose responses and subcellular NR4A1 expression. Related to Figure 7.**

**A** Dose-responses of DG aNSPCs and SEZ aNSPCs to different C-DIM5 and C-DIM8 concentrations. The % Trypan Blue+ cells was determined after 48 h as a read-out for cell death induced by the compounds relative to the vehicle control DMSO. 15 µM C-DIM5 and 15 µM C-DIM8 were established as the highest dosages, which did not elevated cell death relative to the vehicle control DMSO. n = 4 biological replicates / group.

**B** Confocal images of DG and SEZ aNSPCs transduced with a GFP-control (CAG-GFP) or FOXG1-overexpressing (CAG-FOXG1-IRES-GFP) retrovirus and treated for 48 h with DMSO/C-DIM5/C-DIM8 (each 15 µM). Immunohistochemistry for NR4A1 in red. DAPI in blue. Scale bar = 10 µm.

**C** Analysis of the Corrected Total Cell Fluorescence (CTCF) of NR4A1 in the cytoplasm or the nucleus detected a significant increase in nuclear NR4A1 signal and significant decrease in cytoplasmic NR4A1 signal after treatment with C-DIM5 (15 µM for 48 h) in DG and SEZ aNSPCs under control conditions and upon FOXG1 overexpression. C-DIM8 treatment (15 µM for 48 h) significantly decreased the cytoplasmic NR4A1 signal, while nuclear NR4A1 signal was not altered in DG and SEZ aNSPCs under control conditions and upon FOXG1 overexpression. n = 4 biological replicates / group.

Data represented as mean ± SEM; Welch’s t-test was used to determine significance if not indicated otherwise; *p<0.05, **p<0.01 and ***p<0.001.

**Supplementary Reference**

1. Xuan S, Baptista CA, Balas G, Tao W, Soares VC, Lai E. Winged helix transcription factor BF-1 is essential for the development of the cerebral hemispheres. *Neuron* 1995; **14**(6)**:** 1141-1152.
